# Cloning, expression, and one-step purification/immobilization of two carbohydrate-binding module-tagged alcohol dehydrogenases

**DOI:** 10.1186/s13036-022-00295-8

**Published:** 2022-06-28

**Authors:** Mario Benito, Ramón Román, Garazi Ortiz, Antoni Casablancas, Gregorio Álvaro, Gloria Caminal, Gloria González, Marina Guillén

**Affiliations:** 1 Bioprocess Engineering and Applied Biocatalysis Group, Department of Chemical Biological and Environmental Engineering, Universitat Autònoma de Barcelona, 08193 Bellaterra, Spain; 2 Institute of Advanced Chemistry of Catalonia, IQAC-CSIC, Jordi Girona 18-26, 08034 Barcelona, Spain

**Keywords:** Carbohydrate-binding module, *Escherichia coli*, Alcohol dehydrogenase, One-step immobilization/purification, Regenerated amorphous cellulose

## Abstract

**Background:**

The feasibility of biochemical transformation processes is usually greatly dependent on biocatalysts cost. Therefore, immobilizing and reusing biocatalysts is an approach to be considered to bring biotransformations closer to industrial feasibility, since it does not only allow to reuse enzymes but can also improve their stability towards several reaction conditions. Carbohydrate-Binding Modules (CBM) are well-described domains involved in substrate binding which have been already used as purification tags.

**Results:**

In this work, two different Carbohydrate-Binding Modules (CBM3 and CBM9) have been successfully fused to an alcohol dehydrogenase from *Saccharomyces cerevisiae*, which has been produced in bench-scale reactor using an auxotrophic M15-derived *E. coli* strain, following a fed-batch strategy with antibiotic-free medium. Around 40 mg·g^− 1^ DCW of both fusion proteins were produced, with a specific activity of > 65 AU·mg^− 1^. Overexpressed proteins were bound to a low-cost and highly selective cellulosic support by one-step immobilization/purification process at > 98% yield, retaining about a 90% of initial activity. Finally, the same support was also used for protein purification, aiming to establish an alternative to metal affinity chromatography, by which CBM9 tag proved to be useful, with a recovery yield of > 97% and 5-fold increased purity grade.

**Conclusion:**

CBM domains were proved to be suitable for one-step immobilization/purification process, retaining almost total activity offered. However, purification process was only successful with CBM9.

## Background

The numerous advantages of enzyme biocatalysis led to its increased utilization in the synthesis of a vast scope of target molecules over the last decades, especially when processes with high regio- and enantio-selectivity are required [1, 2]. However, many industries are still reluctant to adopt enzyme biocatalysts in large-scale production processes due to the fact that the conditions under which these enzymes are expected to operate in industry are also very often far from those found in nature, further affecting their activity and stability [3].

Moreover, since the performance of biocatalyst and its production contribute to the final operating cost, it is advisable to explore diverse strategies in order to improve biocatalyst yield, what can be achieved via targeted protein engineering, biocatalyst modification, and improved heterologous production [4, 5], as well as through the implementation of process intensification options on enzyme purification and enzyme recycling [6].

In that context, *Escherichia coli* is probably the most widely used prokaryotic system for the synthesis of heterologous proteins thanks to their many advantages including high growth rates and high production yields achieved by using inexpensive culture media, wide knowledge of its metabolism and genome and its easy transformation ability with exogenous DNA, among others [7, 8, 9]. Several *E. coli* strains have been established in industrial bioprocesses, mainly *E. coli* BL21 and K-12 derived strains [10].

Besides, another approach commonly used to bring biotransformations closer to industrial feasibility is biocatalyst immobilization, which does not only allow to reuse enzymes but can also improve their stability towards several reaction conditions such temperature, pH or solvents, thereby increasing the biocatalyst yield [11]. Nowadays, a wide scope of biocatalyst immobilizations are well-described and different supports are commercialized on that purpose [6, 12]. In addition, the use of immobilized enzymes can offer operational advantages by enabling the use of packed-bed reactors, what can lead to simplified product recovery processes [13, 14]. Using purified and isolated biocatalysts instead of crude cell broth it is also an advisable strategy, preventing secondary undesired reactions and reducing the implementation of excessive downstream steps.

In this context, many affinity interactions that are used to purify enzymes can also be applied to immobilize them, but probably one of the most typically used is immobilized-metal affinity chromatography (IMAC), in which the ligand is a metal chelator to which a metal ion has been bound [15]. Supports with Co^2+^ or Ni^2+^ ions are capable to bind histidine-bearing peptides, especially those proteins with a poly-histidine tag fused to one of their ends [16]. However, these kinds of resins used to purify or to immobilize histidine-tagged proteins contribute significantly to the final downstream cost, what brings to contemplate other alternatives, especially for industrial-scale processes. Additionally, the presence of metal ions in final product is not accepted in processes focused on pharma or food industries.

That said, Carbohydrate-Binding Modules (CBM) are well-described as non-catalytic domains involved in substrate binding of many carbohydrate-active enzymes [17], which have shown high affinity for a wide range of polysaccharides. Currently, thousands of CBMs have been divided into 88 different families based on amino acid sequence, binding specificity, and structure [18].

Many CBMs have been already used as purification tags not only because of their highly specificity but for other reasons too, since CBMs confer an enhanced protein folding and solubility, as well as increased protein overexpression yields [19, 20, 18]. The most frequently used CBMs are those that bind to cellulose, especially CBM3 [21, 22, 23] and CBM9 [24, 25, 20], but many others have been studied [26, 27].

In this study, one CBM3 domain from *C. thermocellum* and one CBM9 domain from *T. maritima* have been fused to alcohol dehydrogenase 1 from *S. cerevisiae* (*Sc*ADH), and both constructs have been overexpressed with an auxotrophic *E. coli* strain [28, 29]. The first objective of this work was to produce both fusion proteins by using minimum culture media in a bench-scale reactor. In order to determine if the *E. coli* strain could produce CBM-fused proteins as efficiently as histidine-tagged, production parameters such volumetric productivity, titre and specific mass and activity production were compared.

Moreover, specific activity was compared with the histidine-tagged version aiming to evaluate the effect of the CBM fused tags to enzyme’s catalytic capacity. This study was performed to establish a one-step purification/immobilization process for the two CBM-fused enzymes, based on affinity interactions between CBMs and cellulosic supports, as an alternative of Ni^2+^-NTA resins. Immobilization parameters have been determined for both candidates, and the stability of immobilized derivatives has been analyzed too.

Finally, the feasibility of CBM-fused *Sc*ADH purification by FPLC chromatography has also been assessed, aiming to recover highly purified soluble target proteins, as another possibility different from enzyme immobilization, which would determine the versatility of the cellulose as a support for both i) one-step immobilization/purification and ii) enzyme purification by cellulose affinity chromatography.

## Materials and methods

### Reagents and materials

All reagents were purchased from Sigma Aldrich® (St. Louis, MO, USA) and all molecular biology reagents, FastDigest enzymes and purification kits were purchased form Thermo Scientific™ (Waltham, MA, USA), unless otherwise stated. Avicel® PH-200 microcrystalline cellulose sample was kindly donated by DuPont™ N&B (New York, NY, USA).

### Bacterial strains and plasmids

*E. coli* DH5α strain (Invitrogen, Thermo Scientific) was used for vector propagation, cloning and gene expression. *E. coli* M15Δ*gly*A strain [28] was used for protein overexpression. *Saccharomyces cerevisiae* alcohol dehydrogenase 1 (*Sc*ADH, EC 1.1.1.1) DNA sequence was obtained from a pBAD vector shared by Marco W. Fraaije group at Groningen University (RUG, Netherlands). 6xHis-ADH and CBM-ADH fusion proteins were cloned into a vector named pVEF, derived from an in-house developed plasmid [29], which contains among other features a T5 promoter, a lac operator (inducible with IPTG) and an ampicillin resistance-coding gene (AmpR). Plus, it contains the LacI-*gly*A cassette, regulated by a constitutive promoter (J23110). DNA fragments corresponding to CBM3 from CtCipA and CBM9 from TmXyn10A were synthesized by GenScript Biotech (Piscataway, NJ, USA) considering codon usage optimization for *E. coli*, and were delivered inside a pUC57 plasmid. *Sc*ADH DNA sequence was already optimized.

### DNA amplification

All DNA sequences were amplified by PCR using a Phusion Flash High-Fidelity PCR Master Mix. PCR reactions were performed in an Applied Biosystems™ MiniAmp™ Thermal Cycler (Thermo Fisher), and amplified DNA was purified with a GeneJET PCR purification Kit, according to the manufacturer’s instructions. Common PCR was performed for 6xHis-ADH amplification, whereas for cloning CBM-tagged enzymes, it was required to perform a two-step overlap extension PCR. Between CBMs and ADH sequences it was included a 36-nucleotide linker fragment whose DNA sequence was 5′ AGCGCGGGCAGCAGCGCG GCGGGCAGCGGCAGCGGC 3′. Primers used in PCR reactions are listed in Table 1. Annealing temperature depended on the design of the primers and it lasted 30 seconds. Elongation temperature was fixed on 72 °C and time varied depending on the length of the fragment (30 second per each 1000 bp). PCR reactions were checked by agarose gel electrophoresis using 5 mM Lithium acetate buffer (LA) and SYBR Safe as staining reagent.

**Table 1:** List of oligonucleotides used as primers in polymerase chain reactions of histidine-fused enzymes

Name (sense)	5′ to 3′ DNA sequence	Size (bp)	Tm (°C)	GC (%)
his-ADH (Fw)	AGGAGAAATTAACCC**ATG**GGCAGCAGC**CAT**CAT	33	68	48
*Sc*ADH (Rev)	CTAATTAAGCTTCCCTTATTTAGAAGTGTCAACAACG	37	61	35
CBM3-linker (Fw)	AGGAGAAATTAACCC**ATG**AACCTGAAAGTGGAA	33	63	39
CBM9-linker (Fw)	AGGAGAAATTAACCC**ATG**GTGGCGACCG	28	66	54
Linker-*Sc*ADH (Rev)	**GGATAGACATGCCGCTGCCG**CT	22	66	64
Linker-*Sc*ADH (Fw)	**CGGCAGCGGCATGTCTATCC**CAGAAACTCA	30	68	57

Legend: Tm, melting temperature in °C; GC, guanine-cytosine content in %. Overlapping region between the two DNA fragments of each construct pointed out in bold (green), first codifying codon pointed out in bold and stop codon underlined, codon corresponding to first histidine residue pointed out in bold (blue). Fw, forward, Rev., reverse.

### Enzyme cloning

Fusion proteins were cloned into a previously SmaI linearized pVEF vector using a variation of the sequence- and ligation-independent cloning (SLIC) method [30, 31]. Briefly two separate T4 DNA polymerase reactions for linearized vector and insert were carried out containing in 10 μL final volume, 40-50 ng of either linearized vector DNA or the corresponding insert DNA in 1:2 M ratio. Reaction buffer was composed of 200 mM Urea, 20 mM DTT, 33 mM Tris-acetate (pH 7.9), 10 mM Magnesium acetate, 66 mM potassium acetate, 0.1 mg·mL^− 1^ BSA and 2.5 U of T4 DNA polymerase. Both were incubated at 12 °C for 10 minutes. Ethylenediaminetetraacetic acid (EDTA) (45.5 mM) was added in each tube to stop the reaction and the tubes were incubated at 75 °C for 10 minutes afterwards. The two reactions were then mixed and annealed progressively using a temperature decrease ramp of 2.4 °C·min^− 1^ for 20 minutes, from 72 to 24 °C.

### *E. coli* transformation

100 μL of *E. coli* DH5α competent cells were transformed by heat-shock with 10 μL of SLIC product. Transformed clones were selected using Luria–Bertrani medium (LB)-agar plates supplemented with ampicillin 100 mg·L^− 1^ (incubated overnight at 37 °C in a Sanyo MIR-154 incubator). Transformations were confirmed by colony-PCR and by plasmid restriction pattern with XbaI enzyme (37 °C, 30 minutes). For the latter experiments, plasmid was recovered from coli cells using a GeneJET Plasmid Miniprep Kit, following manufacturer’s procedure. Eventually, constructs were sequenced and verified using an ABI3130XL automated DNA sequencer device (Applied Biosystems, CA, USA) at IBB facilities (Institute of Biotechnology and Biomedicine, UAB, Spain).

Same transformation protocol was followed to transform *E. coli* M15Δ*gly*A competent cells. Aiming to establish a ready-to-use cell bank cryostock with the most productive *E. coli* M15Δ*gly*A colonies, well plate cultures were seeded with transformed colonies from LB-agar plates. Protein production screening (2 mL LB medium, 24 °C, 24 h, 140 rpm, 0.4 mM IPTG) was carried out and more productive candidates were used to generate the final stock (MD medium, 25% v/v glycerol, − 80 °C) after three cycles of adaptation to minimum medium (see section 2.7.1).

### Media composition

LB medium was used for molecular biology experiments and for preliminary production studies in well plates. A defined minimum medium (DM) with glucose as carbon source was used for cryostock generation and for protein overexpression at bioreactor scale. DM composition has been already described [29], as well as feeding medium for fed-batch phase. No antibiotic was used after molecular biology experiments.

### Cultivation conditions

#### Shake-flask cultures

Before generating cell bank stocks, M15Δ*gly*A colonies were adapted to DM by performing a three-step adaptation cultures in 100 mL Erlenmeyer flasks with 30 mL of DM (overnight at 37 °C and 140 rpm of agitation). Pre-inoculum cultures were prepared with 15 mL of DM plus 100 μL of the resulting cell cryostocks, in 50 mL Erlenmeyer flasks (37 °C, 140 rpm, overnight). Inoculum cultures started at an OD_600_ of 0.2 in duplicate 500 mL Erlenmeyer flasks, using 100 mL of DM per flask (37 °C, 140 rpm). Cultures were kept for 4 to 6 h, until the biomass concentration overtook an OD_600_ of 1.

#### Bioreactor cultures (fed-batch processes)

Fed-batch experiments were performed in an Applikon ez-Control (Applikon Biotechnology®, Delft, Netherlands) equipped with a 2 L vessel, and with PO_2_ and pH probes. Temperature was maintained at 37 °C and pH at 7.0 by adding NH_4_OH 15% v/v and 2 M H_2_SO_4_ solutions. Airflow of 1vvm was applied, and oxygen saturation level was set to a PO_2_ of 60%, controlled through a cascade of stirring (450-1100 rpm) and pure oxygen addition after maximum stirring was reached. Initial batch phase was started by transferring 200 mL of inoculum to 800 mL of DM, with 20 g·L^− 1^ of glucose. Afterwards, once the initial glucose was totally consumed, the substrate limiting fed-batch phase started by adding exponentially the feeding medium through a preprogrammed exponential addition, using the following equation (Eq. (1)):

1 $$\mathrm{F}=\frac{\upmu \ast \mathrm{X}\ast {\mathrm{V}}_0\ast {\mathrm{e}}^{\left(\upmu \ast \Delta \mathrm{t}\right)}}{{\mathrm{Y}}_{\mathrm{X}/\mathrm{S}}\ast {\mathrm{S}}_0}$$

Where F corresponds to the feeding flux (mL·min^− 1^), μ to the set specific growth rate (0.2 h^− 1^), Δt to the time interval in which the feeding flux is applied (1 h), X to the predicted biomass concentration (g·L^− 1^) in the bioreactor at the end of the time interval, V_0_ to the culture volume at the beginning of the time interval, Y_X/S_ to the biomass/substrate yield (set at 0.3 g·g^− 1^) and S_0_ to the concentration of substrate – glucose - in the feeding medium (~ 500 g·L^− 1^).

Induction phase started by adding a pulse of 100 mM IPTG (0.25 mM final concentration) when the culture surpassed a biomass concentration OD_600nm_ ≈ 100, and the whole process ended when cell growth stopped and glucose accumulation was detected, reaching in all cases a final culture volume of approximately 2 L.

### Product recovery

Biomass was separated from the culture media by centrifugation at 7000 rpm for 20 min at 4 °C in an Avanti™ J20 centrifuge (Beckman Coulter, Brea, CA, USA) in 500 mL centrifugation tubes. The pellet was divided into several aliquots and kept frozen at − 20 °*C. prior* to measure the enzymatic activity or purify the enzyme, the aliquot of interest was resuspended in 50 mM Tris−HCl (pH 7.50) buffer and lysed using an OneShot cell disruptor (Constant Systems Ltd., Daventry, UK), for 2 cycles at 1.47 kbar pressure. Cell debris and insoluble fraction was removed by centrifugation at 13000 rpm for 30 min at 4 °C, for subsequent analysis experiments.

### Analytical methods

The biomass concentration was measured in terms of absorbance at 600 nm of wavelength (OD_600_) using a HACH® D3900 (Hach, Loveland, CO, USA) spectrophotometer, diluting the samples in an absorbance of under 0.9. Biomass concentration expressed as dry cell weight (DCW) was calculated considering that 1 OD_600_ unit is equivalent to 0.3 g DCW/L [32] Samples were analyzed for duplicate.

1 mL of culture sample was centrifuged (13,000 rpm, 3 min) and filtered (0.45 μm) to remove biomass. The resulting supernatant was then used for glucose concentration measurement, using an YSI 20170 system (YSI Inc., Yellow Springs, OH, USA). Samples were diluted to a glucose concentration lower than 10 g/L and were analyzed for duplicate.

Total intracellular protein content present in cell lysates were determined with the Bradford method using a Coomassie Protein Assay Reagent Kit (Thermo Scientific) and bovine serum albumin (BSA) as standard, following manufacturer’s instructions. The assays were performed in 96-microwell plates and Multiskan™ FC equipment (Thermo Scientific) was used for the absorbance reading (595 nm). Samples and standard regression points were analyzed for duplicate. The samples were subjected to SDS-PAGE analysis as previously described [33] in order to o determine the percentage of *Sc*ADH enzyme among the rest of intracellular soluble proteins present in the lysates. Protein quantification was performed via densitometry by using Image Lab™ software from Bio Rad.®.

Alcohol dehydrogenase activity present in lysates was determined by following spectrophotometrically the formation of NADH at 340 nm of wavelength with a Cary50Bio UV-visible spectrophotometer (Agilent Technologies, Santa Clara, CA, USA), as a consequence of the enzyme conversion of ethanol to acetaldehyde that requires the presence of NAD^+^ as cofactor. The reaction mixture contained ethanol at 543.6 mM, β- NAD^+^ at 7.5 mM, 20 mM of phosphate buffer (pH = 8.80), and 50 μL of enzyme sample at a final assay volume of 1.5 mL in 1 cm-depth polystyrene cuvettes. One unit of ADH activity was defined as the amount of enzyme required to catalyze the conversion of 1 μmol of NAD^+^ to NADH per minute at 25 °C, being the molar extinction coefficient of β-NADH of 6.22 mM^− 1^·cm^− 1^. Activity measurements of all samples analyzed were carried out for triplicate.

### Immobilization of CBM to cellulosic support

RAC cellulose was prepared from Avicel®-PH101 (Sigma) and Avicel®-PH200 (DuPont) by following a well-described procedure [34], consisting in acid treatment of microcrystalline cellulose at low temperature.

Immobilization experiments were performed with 9 mL of cell lysate containing CBM3- or CBM9-fused ADH and 1 mL of filtered RAC obtained from Avicel® PH-101. Immobilization processes were carried out at room temperature (24 °C) with roller bottles and all experiments were done for triplicate. Retained activity (RA) and immobilization yield (IY) were calculated using the following equations (Eq. (2) and (3), respectively):

2 $$\mathrm{RA}\ \left(\%\right)=\frac{Asus- Asn}{\mathrm{Ai}}\cdot 100$$

3 $$\mathrm{IY}\ \left(\%\right)=\frac{Ai- Asn}{\mathrm{Ai}}\cdot 100$$

Where A_i_ corresponds to initial enzyme activity offered, A_sn_ to remaining enzyme activity measured in supernatant at the end of experiment and A_sus_ to enzyme activity of the suspension (supernatant plus support) measured at the end of experiment. Several samples were taken along the experiment time and enzyme activity was measured for triplicate. Error bars correspond to standard error of all activity measurements. Immobilized derivatives were separated from cell lysate by vacuum filtration and were resuspended in fresh 50 mM Tris-HCl pH 7.50 buffer.

For maximum load capacity assessment, cell lysate volumes used were higher than 9 mL since greater enzyme amounts were required to determine the maximum AU value that can be immobilized in 1 mL of cellulosic support.

### FPLC purification of CBM-fused enzyme

For purification of CBM-fused ADH enzyme, a fast protein liquid chromatography (FPLC) process based on previous reports [24] was carried out using an ÄKTA™ pure 150 equip from Cytiva (Uppsala, Sweden) with a Pharmacia XK-16/20 column packed with 10 mL of Avicel® PH200-derived RAC. A 10 mL sample of the clarified cell lysate was loaded at 0.5 mL·min^− 1^ onto the column - previously conditioned with 2 column volumes (CV) of 50 mM Tris-HCl buffer, pH 7.50 -. The column was first washed with 5 CV of 200 mM NaCl in 50 mM Tris-HCl buffer, pH 7.50 (5 mL·min^− 1^) and with 3 CV of 50 mM Tris-HCl buffer, pH 7.50 (5 mL·min^− 1^) afterwards. The bounds between CBMs and RAC were desorbed with 3 CV of 2 M glucose in 50 mM Tris-HCl buffer, pH 7.50 (2 mL·min^− 1^). Process was carried out at room temperature (24 °C) and all fractions were collected and subsequently analyzed by Bradford assay, by SDS-PAGE electrophoresis and by activity assay.

## Results and discussion

### Histidine- and CBM-tagged enzyme cloning and overexpression

Molecular biology experiments led to the obtaining of *E. coli* M15Δ*gly*A cells capable to overexpress satisfactorily the histidine-tagged alcohol dehydrogenase from *S. cerevisiae* as well as CBM3-*Sc*ADH and CBM9-*Sc*ADH fusion proteins, not only in complex LB medium but also in minimum defined medium.

SLIC technique turned out to be an effective and cost-saving method, since DH5α cell colonies were grown in LB-agar plates in all cases after cell transformation process. DNA sequencing confirmed the correct cloning with no mutations detected in any case.

**Fig. 1:**
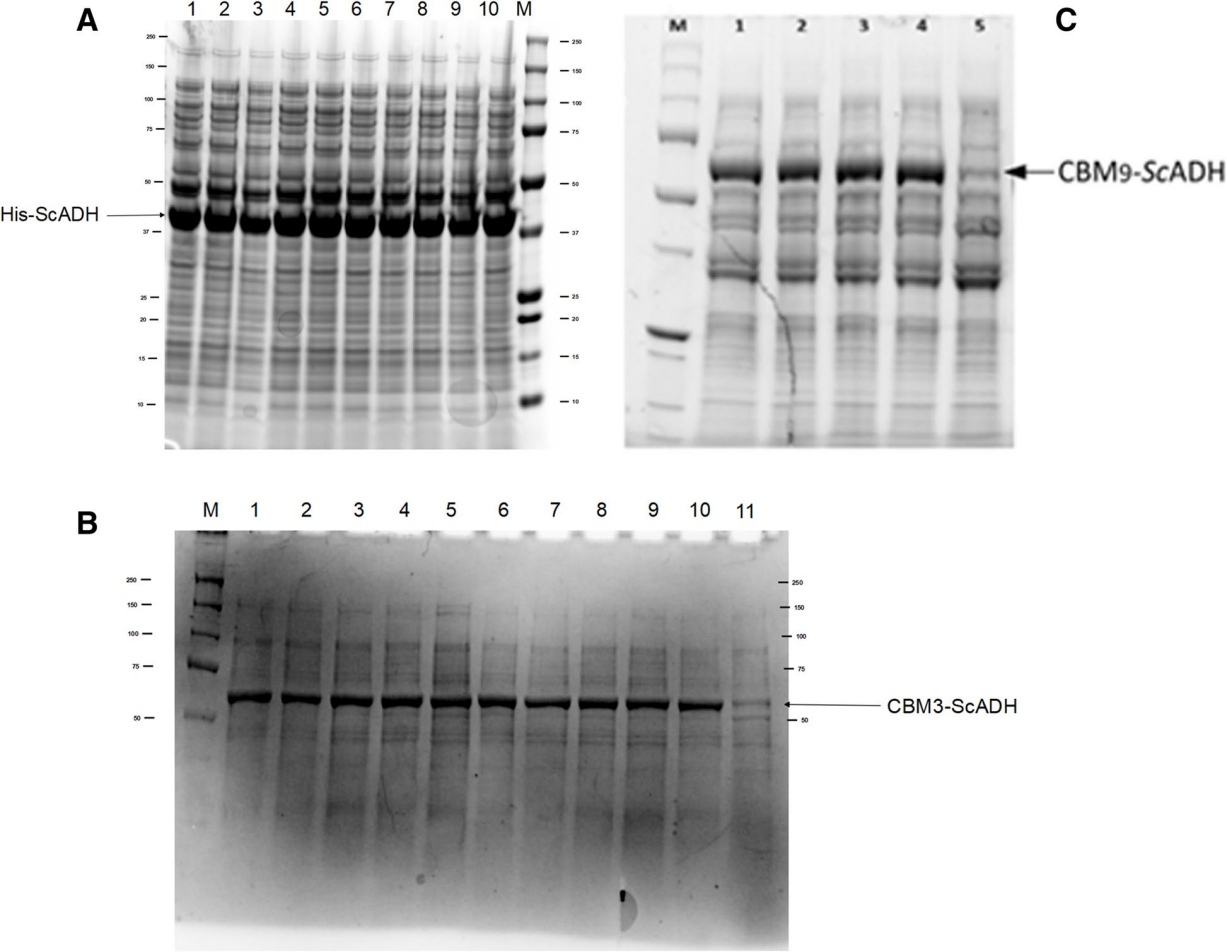
SDS-PAGE of *E. coli* M15Δ*gly*A final samples from well plate cultures in LB medium. **A**: His-*Sc*ADH. Lane M: molecular weight standard (kDa); lanes 1 to 10: induced cultures. **B**: CBM3-*Sc*ADH. Lanes 1 to 10: induced cultures; lane 11: negative control. **C**: CBM9-*Sc*ADH. Lanes 1 to 4: induced cultures; lane 5: negative control. Culture conditions: 2 mL LB, 24 °C, 24 h, 140 rpm, 0.4 mM IPTG. His-*Sc*ADH (37 kDa), CBM3-*Sc*ADH (55 kDa) and CBM9-*Sc*ADH (64 kDa) corresponding bands indicated with arrows

*E. coli* M15Δ*gly*A overexpression screening (Fig. 1) was useful to determine that all clones were able to produce the target proteins. Nevertheless, the percentage of recombinant protein with respect to total protein content (determined by SDS-PAGE) varied quite significantly between the different constructs, from 26% for His-*Sc*ADH (Fig. 1A) to 34 and 38% of relative band intensity for CBM3-*Sc*ADH (Fig. 1B) and CBM9-*Sc*ADH (Fig. 1C), respectively. This increased overexpression levels for CBM-fused variants could somehow reveal the beneficial role of these kind of protein domains, as previously reported [19].

Minor differences were observed between clones of the same variant, and the colony of each construct which showed the greater overexpression level - according to SDS-PAGE -was picked to generate the cell bank for the subsequent production processes, being these colonies those corresponding to Fig. 1A lane 4, Fig. 1B lane 6 and Fig. 1C lane 3, respectively.

### Enzyme production at bioreactor scale

Aiming not only to compare production yields between the three constructs but also to determine if the N-terminal-fused CBMs domains affected negatively to *Sc*ADH catalytic activity, the three recombinant proteins were produced in a 2 L bench-scale reactor, using minimum and free-antibiotic media with glucose as carbon source as described.

**Fig. 2:**
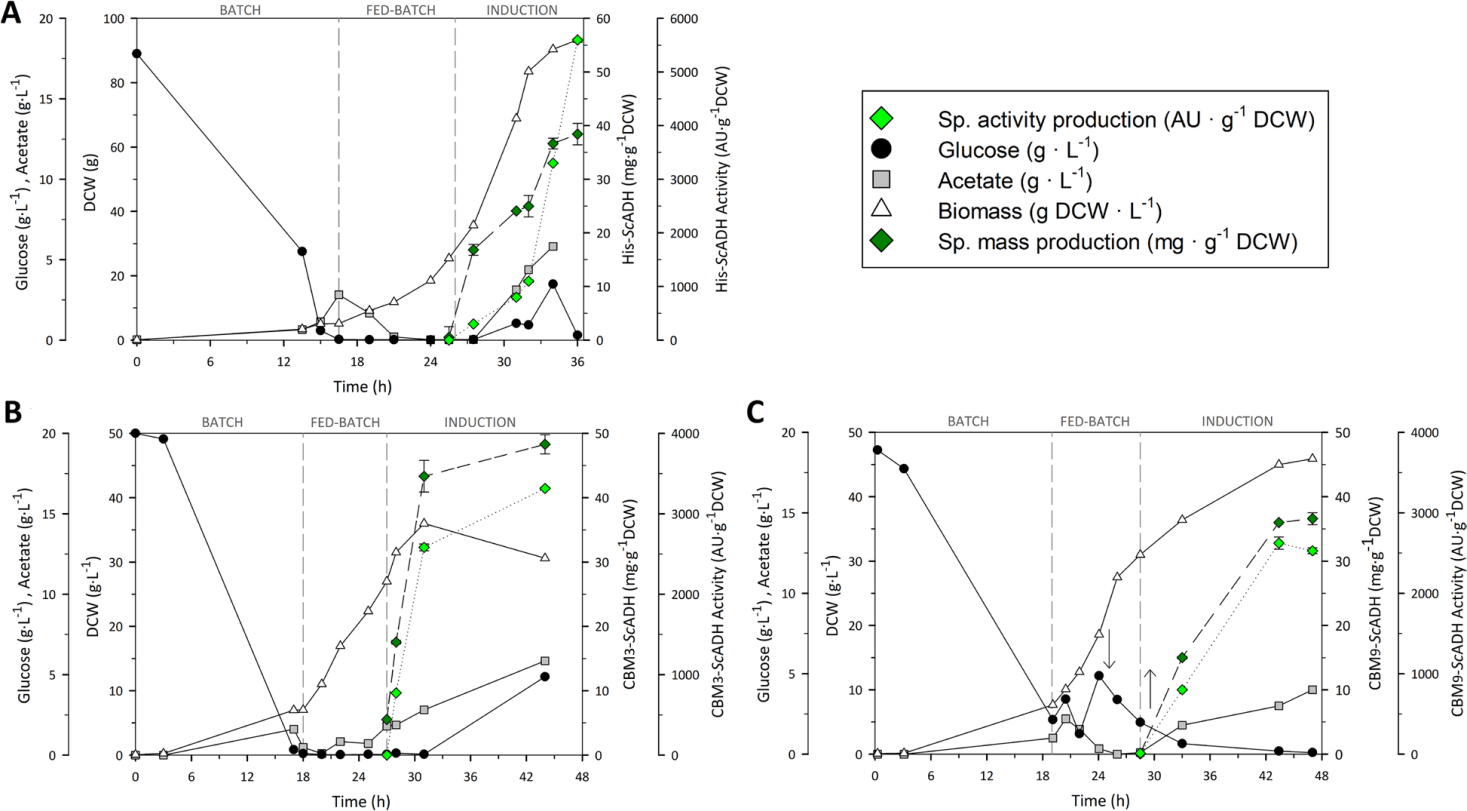
*E. coli* M15Δ*gly*A fed-batch cultures. Batch, fed-batch and induction ([IPTG] 0.25 mM) phases indicated. Culture conditions: 37 °C, pH 7.0, 450-1200 rpm, 60% PO_2_, 0.25 mM IPTG. **A**: His-*Sc*ADH, **B**: CBM3-*Sc*ADH and **C**: CBM9-*Sc*ADH. Arrows indicate the stop of the feeding (↓) and the resume of the feeding (↑)

Bioprocess parameters such biomass, substrate, enzyme specific activity and specific mass production were analyzed along the three processes (Fig. 2).

Regarding the development of the three processes, batch phase lasted for about 16 to 18 hours, with an approximate biomass/substrate yield close to the predicted value of 0.3 g of biomass per gram of glucose. Fed-batch phase duration was also similar in all cases, reaching the desired biomass concentration for culture induction in about 8 to 10 hours of exponential growth. Specific growth rate (μ) of 0.18 h^− 1^ was calculated during exponential feeding addition, except in CBM3-*Sc*ADH production case (Fig. 2B), in which a μ of 0.15 h^− 1^ was determined. The differences between the pre-set μ in feeding addition and the experimentally measured μ could be due to the coefficient of cell maintenance [35], which was not considered. Besides, CBM9-*Sc*ADH production process (Fig. 2C) experienced an accumulation of glucose at the early stage of fed-batch phase, probably caused by an acetate accumulation at the end of batch phase. In that case, feeding was stopped until glucose concentration decreased below 5 g·L^− 1^ and acetate was completely exhausted.

In all cases, target protein overexpression mechanism was strongly repressed, since first cell lysate samples – corresponding to the moment prior to induction - showed negligible enzyme activity, which increased quite notably in later samples. Protein overexpression induction caused in all cases a metabolic imbalance that led to the accumulation of acetate, glucose, and the subsequent decrease in cell growth, as expected. Production parameters, including the activity units per mol of enzyme (specific activity) were determined for the three cases and were listed in Table 2.

**Table 2:** Production parameters of *Sc*ADH using *E. coli* M15Δ*gly*A; comparison between the three different N-terminal-fused tags

	(6x) Histidine	CBM3	CBM9
Enzyme activity (AU·L^−1^)	2.83·10^5^	1.02·10^5^	1.16·10^5^
Enzyme titer (mg·L^−1^)	1940	1550	1780
Volumetric productivity (AU·L^−1^·h^− 1^)	7870	2300	2520
Volumetric productivity (mg·L^−1^·h^− 1^)	54	35.3	38.6
Specific production (mg·g^−1^DCW)	38.4	50.7	36.6
Specific production (AU·g^−1^DCW)	5600	3320	2390
Specific activity (AU·mg^−1^)	145.7	65.4	65.3
Specific activity (AU·μmol^−1^) *	5380	3580	3860

* Considering a molar weight of 37 kDa for His-ScADH, 55 KDa for CBM3-ScADH and 64 kDa for CBM9-ScADH

Even if similar total amounts of target protein were produced, obtaining 3.58 g of His-*Sc*ADH, 3.13 g of CBM3-*Sc*ADH and 3.48 g of CBM9-*Sc*ADH, specific mass production (mg·g^− 1^DCW) of CBM3-fused enzyme was slightly higher than histidine- and CBM9-fused ADH (Table 2). This increase was mainly caused by the difference of total biomass obtained in each case, being similar for histidine- and CBM9-fused variants (93 and 90 g DCW, respectively) but much lower for CBM3-tagged enzyme (60 g DCW).

Minor differences were observed among the constructs for target protein overexpression levels, oscillating from 10 to 12%. However, higher values were observed in LB screening experiments, mainly due to temperature shift from well-plate cultures (24 °C) to bench-scale reactor (37 °C); temperature is a well-known and well-described key parameter in recombinant protein production, where greater fraction of synthetized protein tends to fold correctly rather than generate insoluble inclusion bodies at lower culture temperatures because metabolic imbalance produced by a strong protein overexpression induction is tightly affected by temperature [36]. This hypothesis reveals that it still exists room for process optimization, although that was not the point in that case.

Overall, considering that one of the main objectives was the assessment of the possible affectation of CBMs domains to alcohol dehydrogenase’s functionality, specific activity (AU·μmol^− 1^) of the three variants were determined (Table 2), for which histidine-fused version was 1.5-fold higher than CBM-fused ones. Despite the significant activity loss, the fused CBM domains do not seem to negatively affect the catalytic capability of *Sc*ADH enzyme, since the resulting polypeptides are functional and total produced activity values still fluctuate inside the same magnitude order.

### Immobilization of CBM-fused proteins

Aiming to characterize the affinity of CBM domains towards cellulose, CBM-fused *Sc*ADH enzymes were immobilized to a RAC cellulose support. The characterization was carried out by loading approximately 30 AU·mL^− 1^ support, where no diffusional limitations were observed.

**Fig. 3:**
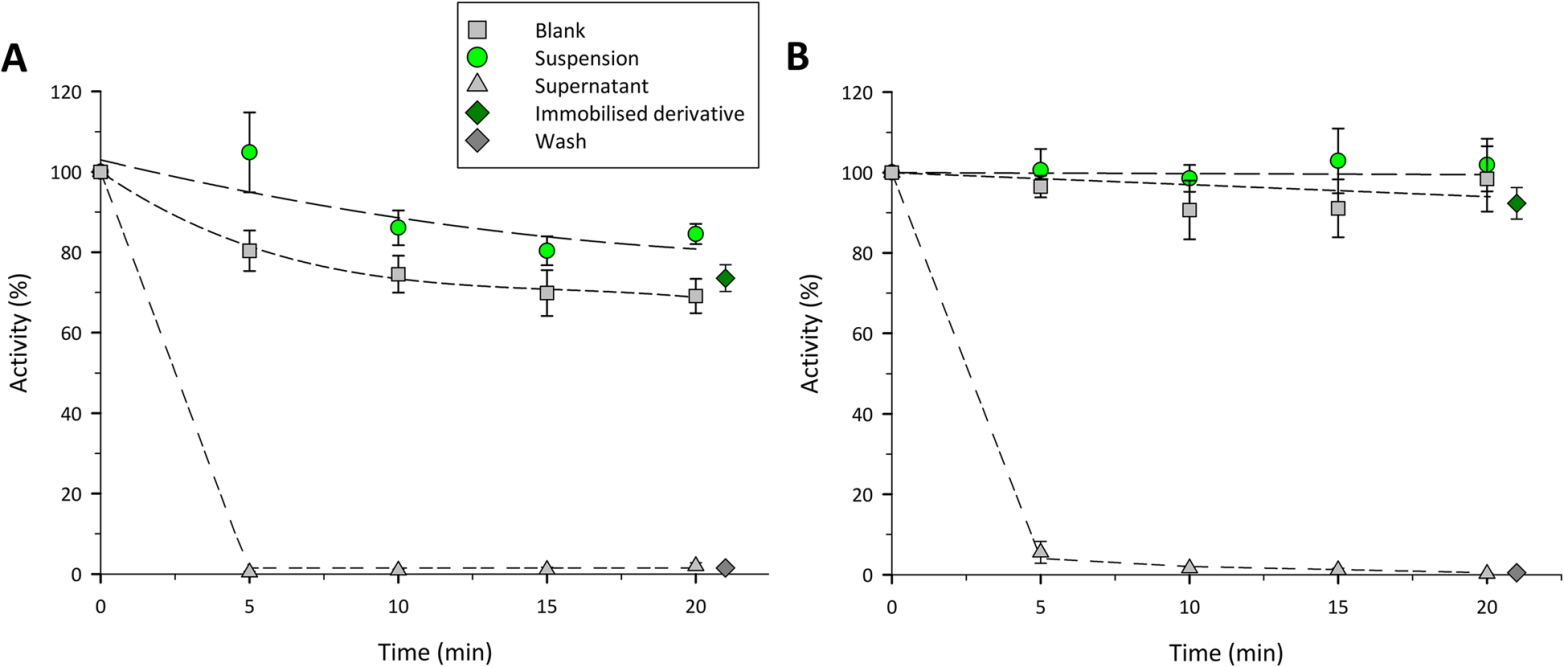
Alcohol dehydrogenase activity along the immobilization processes of CBM3-*Sc*ADH (**A**) and CBM9-*Sc*ADH (**B**) fusion proteins with 1 mL RAC support

First batch experiments (Fig. 3A and B) showed the high affinity of both carbohydrate-binding modules towards cellulose, achieving an almost total binding of target protein after 5 minutes of incubation (Table 3, Fig. 3).

**Table 3:** Immobilization parameters of CBM-fused *Sc*ADH proteins onto RAC support

Tag	Total protein offered (mg)	Enzyme offered (mg)	Enzyme offered (AU)	RA (%)	IY (%)	Recovered activity (%)
CBM3	2.54 ± 0.04	0.37 ± 0.02	29 ± 0.5	86.1 ± 2.2	98.4 ± 1.3	73.6 ± 3.3
CBM9	2.37 ± 0.3	0.22 ± 0	14.2 ± 4.5	97.7 ± 1.7	99.7 ± 0.1	92.4 ± 3.9

Legend: Experiment conditions: 24 °C, pH 7.5, 1 mL RAC support, roller agitation. RA, retained activity and IY, immobilization yield.

Results also showed a slight deactivation of CBM3-fused *Sc*ADH due to the immobilization process as can be observed in suspension and blank activity profiles (Fig. 3A). This fact led to higher retained activities for CBM9-fused enzyme (97.7%) compared to the CBM3-fused *Sc*ADH (86.1%). In addition, recovered activity obtained once the immobilized derivatives were washed was almost 20% higher when CBM9-tag was used.

Regarding the mass balances, in both cases was demonstrated the high specificity of the binding between CBM domains and the cellulosic support, since total protein content difference between initial and final supernatant samples were close to the amount of target protein bound to support. On the one hand, CBM3-*Sc*ADH content in supernatant decreased from 14.6 to 0.7%, while final supernatant quantity decreased to 2.12 ± 0.03 mg (83.5% of initial). On the other hand, CBM9-*Sc*ADH presence in lysate decreased from 12.2 to 1.1%, recovering an 89.1% of total protein content in final supernatant samples (2.11 ± 0.14 mg).

**Fig. 4:**
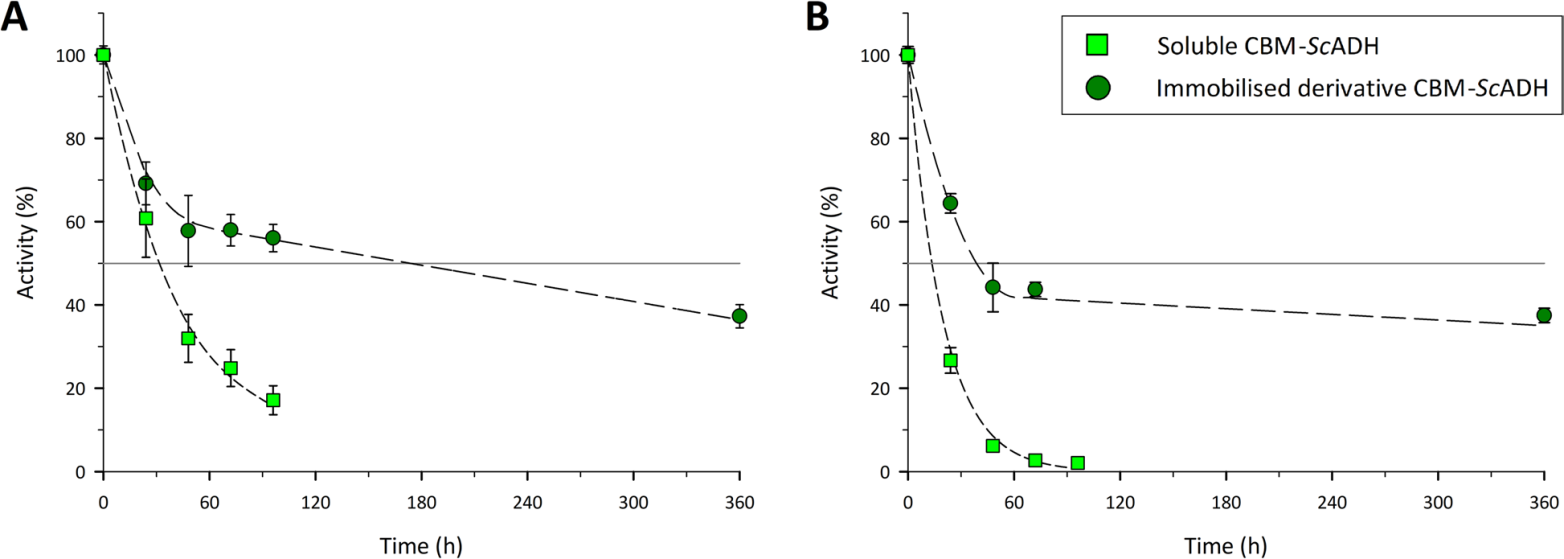
Alcohol dehydrogenase stability for CBM3-*Sc*ADH (**A**) and CBM9-*Sc*ADH (**B**) under refrigeration storage conditions (4 °C). Experiments conditions: 50 mM Tris-HCl buffer pH 7.50, 24 °C, roller agitation. Error bars correspond to standard error of three replicates

These results validate the CBM-tagged enzymes as a promising system for one step purification/immobilization process thanks to i) the high specificity of CBM domains towards RAC compared to the other proteins present in *E. coli* lysates and ii) the high retained activities obtained in the final immobilized derivatives. In order to compare the enzyme storage stability between soluble and immobilized derivatives, samples were kept under refrigeration (4 °C) and suspension activity was measured along time (Fig. 4), revealing that the immobilization process allowed a 2.9-fold increase of half-life - from 13.5 to 38.7 hours - for CBM9-ScADH (Fig. 4B) and 5.5-fold increase for CBM3-ScADH – from 31.6 to 173.6 hours – (Fig. 4A). Despite CBM9-fused protein showed a faster loose of activity than CBM3-*Sc*ADH (both soluble and immobilized), the two immobilized derivatives kept a final relative activity of almost a 40% a fortnight after immobilization experiments were carried out.

Once again, the obtained results consolidate CBM domains as feasible one-step purification/immobilization tags due to the improvement of enzyme stability once the fusion peptides are bound to the support.

The results obtained are in accordance with other immobilisation techniques found in literature; a 90% of RA was achieved by using carboxymethyl dextran (CMD) coated magnetic nanoparticles (CMD-MNPs) activated with epoxy groups, using epichlorohydrin (EClH) [37], by which a 75% of immobilised activity was maintained after 21 days of storage at 4 °C. Besides, other recent studies performed with ADH enzymes reported lower RA values; a 58 and a 62% of RA were reached for ADH variants from *Artemisia annua and Streptococcus mutans, respectively, that were* immobilised onto agarose resins functionalised with epoxy groups [38, 39].

Therefore, CBM-based immobilisation method stands as a time- and cost-saving immobilisation alternative, which also enables to reach one of the highest immobilisation yields reported so far.

**Fig. 5:**
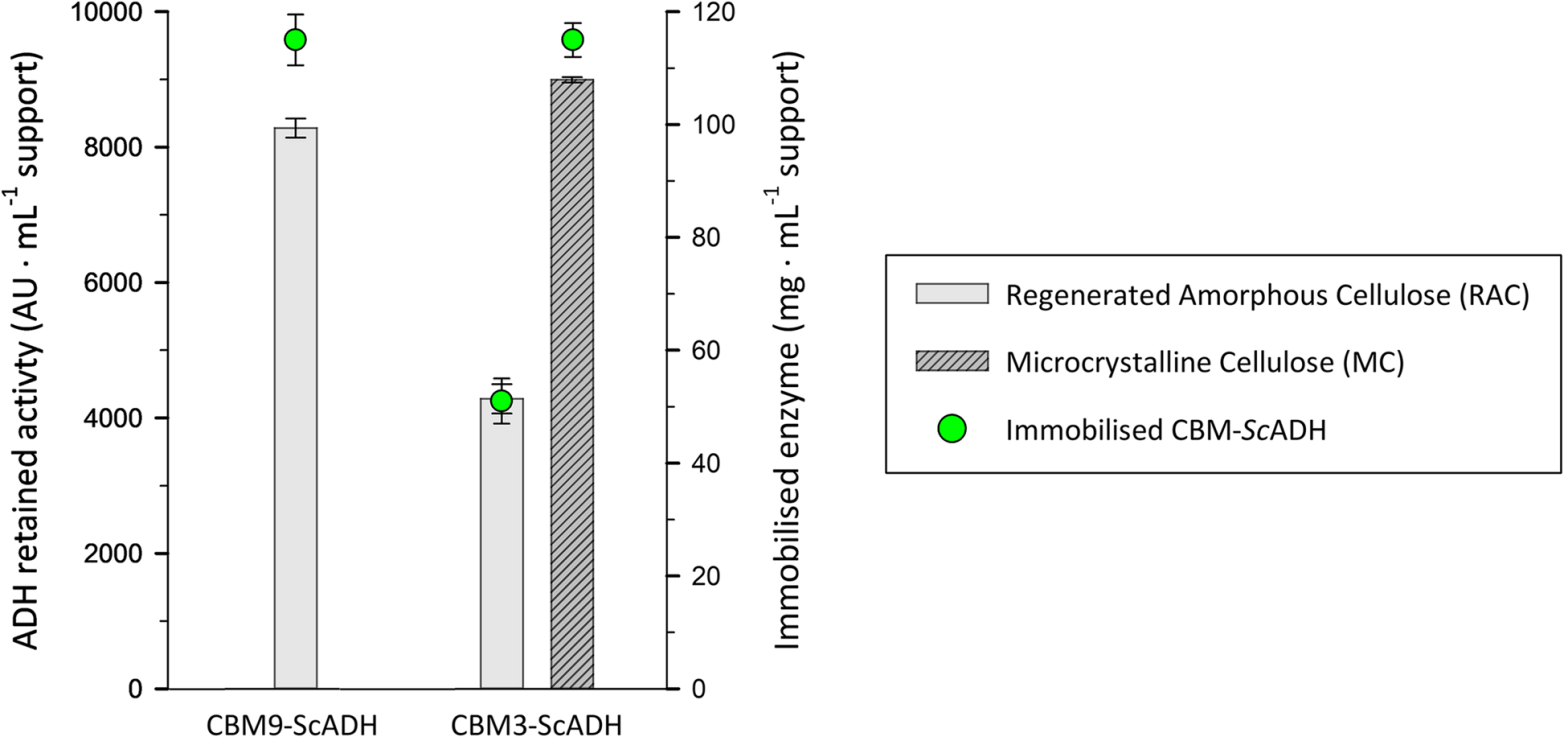
Correlation between alcohol dehydrogenase activity offered and the theoretical retained activity in the support. Experiments conditions: 50 mM Tris-HCL pH 7.50 buffer, 24 °C, roller agitation

The maximum enzyme loading capacity of RAC was analyzed for both CBM-fused enzymes by increasing the offered enzyme quantities (Fig. 5). Both fused enzymes could be successfully immobilized under high loads. However, due to mass transfer limitations, steric hindrances and other possible phenomena commonly associated to highly loaded immobilization supports, retained activity values were underestimated [40]. Thus, RA coefficient previously assessed with no-limiting conditions was used to calculate the theoretical final activity of the high-loaded derivatives by assuming to be equal in all cases, since it is not dependent on enzyme amount [14].

1 mL of RAC support was able to bind up to 7500 ± 275 AU of CBM9-*Sc*ADH enzyme from cell lysate, which would correspond to approximately to 115 ± 4 mg of target protein. For CBM3-*Sc*ADH protein, RA was significantly lower, binding up to 4300 ± 287 AU per RAC mL, corresponding to 66 ± 4 mg of protein.

Nevertheless, considering that diverse CBM families can be established according to the type of compounds by which these domains present a greater binding affinity (see introduction), it was assumable that a higher amount of CBM9-*Sc*ADH fusion protein would be attached to the support, rather than CBM3-*Sc*ADH, since the first family modules are characterized to bind amorphous cellulose while the second ones are not [18].

For that reason, an immobilization experiment with increasing amounts of CBM3-*Sc*ADH was carried out concurrently, but cell lysate was mixed with Avicel® microcrystalline cellulose instead of amorphous cellulose, aiming to corroborate that CBM3 bounds with higher affinity towards non-treated cellulose (Fig. 5). In that sense, unequivocal results were obtained, since 99.8% of IY was achieved when 5000·AU were offered to 1 mL support and 94.5% of IY was measured when 10,000 UA were offered, demonstrating that the most suitable strategy with CBM3 would be using microcrystalline cellulose instead of RAC.

Summarizing, both CBM domains have proved to be useful tags for *Sc*ADH one-step immobilization with cellulosic supports. Even if maximum load capacity of RAC support varied notably depending on the CBM, one positive aspect noticed for both fusion proteins is that enzyme activity remains almost unaltered, making this immobilization method a promising strategy, which also increases storage stability compared with soluble enzyme.

### Use of CBM domains as purification tags

Another possible application that CBMs can provide is their use just as purification tags, based on the reversibility of the bound between cellulose and the protein. This way, several purification methods have already been established, including fast protein liquid chromatography (FPLC) processes [25]. Aiming to compare the efficiency of purification process depending on which CBM is fused to target enzyme, FLCP experiments have been performed, in which enzymes were firstly immobilized to the cellulosic support and were then desorbed with glucose.

However, RAC obtained from Avicel® PH-101 – which has a particle size of approximately 50 μm – was unviable for FPLC performance, because the cellulose bread ended up compacting and column flowthrough collapsed. For that reason, same cellulose support was used but with higher particle size (Avicel® PH-200, ~ 180 μm).

**Fig. 6:**
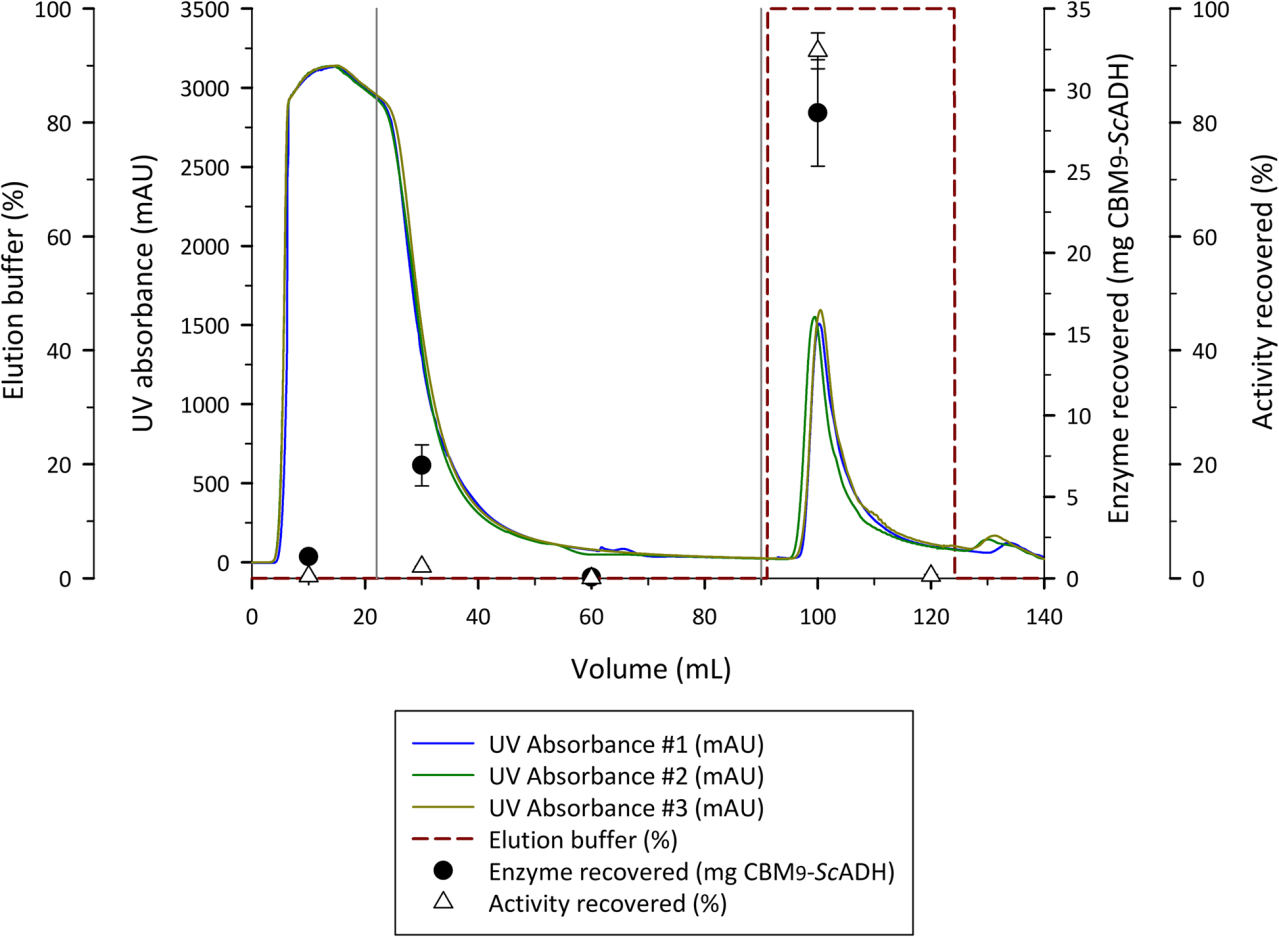
Chromatogram of three consecutive CBM9–*Sc*ADH purifications on pre-treated Avicel® PH-200 support. Experiment conditions: 15 mL column volume, 24 °C, pH 7.50

**Fig. 7:**
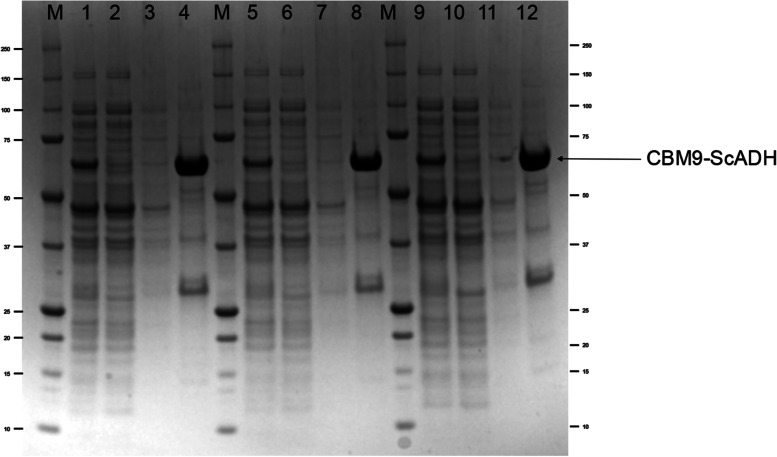
SDS–PAGE of FPLC affinity purifications of CBM9–*Sc*ADH on a 10 mL RAC column. Lanes M: molecular weight standard (kDa); lanes 1, 5 and 9: clarified cell extract prior to column loading; lanes 2, 6 and 10: column flow through; lanes 3, 7 and 11: column wash; lanes 4, 8 and 12: purified CBM9–*Sc*ADH eluted with 50 mM Tris-HCl buffer containing 2 M glucose. CBM9-*Sc*ADH (64 kDa) corresponding band indicated with arrow

For CBM9-*Sc*ADH protein, three affinity purification processes performed consecutively resulted in a 94.7 ± 2.3% recovery of activity in average. A trivial *Sc*ADH fraction within the clarified lysate load was lost in the column flow-through (2.3 ± 0.05%) and no activity was measured at any of the column washes. The resulting chromatogram and the corresponding SDS–PAGE gel documentation of the purification processes is shown in Figs. 6 and 7, respectively, whereas the purification metrics are provided in Table 4.

**Table 4:** FPLC-based *Sc*ADH purification results for CBM9-*Sc*ADH (top) and CBM3-*Sc*ADH (bottom)

Tag	Sample	Total activity (AU)	Total protein (mg)	Recovered activity (%)	Specific activity (AU·mg^**− 1**^)	Purification factor
CBM9	Lysate	4117 ± 99	380.6 ± 8.8	–	10.8 ± 0.5	–
	Eluted	3813 ± 129	35.8 ± 2	94.7 ± 2.3	107.8 ± 6	10.1
CBM3	Lysate	5850 ± 316	440 ± 23	–	13.3 ± 0.4	–
	Eluted	350 ± 1.2	8.8 ± 0.5	6 ± 0	39.7 ± 1.2	3

As expected, a 2 M glucose solution was effective in desorbing all specifically bound target enzyme (Fig. 6), which elutes from the column in a single and clear peak. In addition, it has been proven that RAC support can be reused in consecutive purification batches, since process efficiency and product recovery did not vary significantly among the three experiments performed.

Nevertheless, SDS-PAGE reveals the presence of other proteins in elution fraction (Fig. 7, lanes 4, 8 and 12) – in fact, CBM9-*Sc*ADH purity is about 80% -. Although it has not been determined to what corresponds the band that weights around 30 KDa, it could be a broken fraction of the fusion protein which contains the CBM9 domain, that remains attached to the support until the elution step, but these bands cannot correspond to the CBM fragment (26 kDa) nor the *Sc*ADH enzyme (37 kDa). Besides, purification processes have been performed by adding protease inhibitor (PMSF) to cell extract to precisely prevent the breaking of the fusion protein.

Legend: Avicel® PH-200 RAC used as immobilization support and 2 M glucose (in 50 Tris-HCl buffer) used for protein elution. Experiment conditions: 15 mL column volume, 24 °C, pH 7.50.

On the other hand, CBM3-fused protein affinity purification resulted in a 6% recovery of initial activity (Table 4). Neither the flow-through nor the wash fractions presented any enzyme activity, disregarding then a loss of target protein in previous fractions. Must be stated that elution step resulted in the appearance of a single and clear peak in the chromatogram, but notably smaller than the observed for CBM9-fused variant, meaning that 2 M glucose solution was unable to unbind CBM3 domain from cellulose.

CBM3 domain has been successfully eluted with other compounds such ethyl glycol [22, 23], EDTA [41] or trimethylamine [21]. However, these compounds could not be used in our case of study since they would significantly compromise alcohol dehydrogenase activity.

Protein analysis confirmed what was observed by chromatography, given that elution fraction only contained the 2% of total protein, as opposed to CBM9 case, where 20% of total protein content was recovered in elution fraction.

Moreover, when microcrystalline cellulose was used instead of RAC, only was recovered a 1% of initial activity, which is in accordance with results observed for immobilization process. In other words, the more affinity towards substrate, the stronger bound is established, and the harder to desorb CBM3-fused proteins.

In consequence, these results revealed that CBM3-fused enzymes are suitable for a one-step purification/immobilization process but not applicable, under the tested conditions, for purification process based on the affinity interaction. For that purpose, a CBM9-fused strategy would be a better option since it allows both a single purification process and a one-step purification/immobilization process, and the recovery of a highly active enzyme, which has not been reported in most of the bibliography about CBM domains.

Moreover, it can be also concluded that both CBM3 and CBM9 tags are suitable and cheaper alternative to traditional polyhistidine tag used to purify proteins by IMAC chromatography.

## Conclusions

*Sc*ADH was cloned and overexpressed in *E. coli* following a fed-batch strategy and using antibiotic-free minimum media, not only with a His-tag but with two different CBM domains. Similar product quantities were obtained for the three constructs, but different values were obtained in terms of specific activity, being His-*Sc*ADH 1.5-fold higher than CBM-fused versions.

CBM-tagged variants were proved to be suitable for one-step immobilization/purification process, retaining almost total activity offered. However, maximum load capacity of cellulose support was strongly affected by the nature of the fused CBM. Finally, purification process was only successful for CBM9-fused version, recovering almost total activity.

## Data Availability

The datasets used and/or analysed during the current study are available from the corresponding author on reasonable request.

## Abbreviations

AU: Enzyme activity unit
BSA: Bovine serum albumin
bp: DNA base pair
CDW: Dry cell weight
DNA: Deoxyribonucleic acid
IPTG: Isopropyl β-D-1-thiogalactopyranoside
IY: Immobilisation yield
MCF: Microbial Cell Factory
NAD +: Nicotinamide adenine dinucleotide (oxidized)
NADH: Nicotinamide adenine dinucleotide (reduced)
OD_600_: Optical density at a wavelength (λ) of 600 nm
PAGE: Polyacrylamide gel electrophoresis
PCR: Polymerase chain reaction
PO_2_: Dissolved oxygen
RA: Retained activity
RAC: Regenerated amorphous cellulose
SDS: Sodium dodecyl sulphate
ScADH: Alcohol dehydrogenase from *Saccharomyces cerevisiae*
